# Supplementary material for: Gender diverse people’s psychological wellbeing and identity in the context of gender affirming speech pathology practice: A qualitative study protocol
Source: PLoS One. 2024 Nov 26;19(11):e0311402. doi: 10.1371/journal.pone.0311402 (PMC11594413; doi:10.1371/journal.pone.0311402)
Supplement: S1 Appendix — (PDF) [file pone.0311402.s001.pdf]

Gender diverse people's psychological wellbeing and identity in the context of gender affirming speech pathology practice: A qualitative study protocol

Supporting information

S1 Appendix. Reflexive Thematic Analysis Reporting Guidelines (RTARG) checklist filled in for the planned study[1].

Section 1: The Introduction

**Advice for aspects of the research report/ approach to reporting** [1, suppl]	**Guiding notes and further explanation** [1, suppl]	**Information in the study protocol**	**Addressed in section, page no.**
Background and rationale			
1. Provide a robust context and rationale for the proposed research in the *Introduction*.	Can discuss existing research, theory, and the wider context; the researcher is understood as *entering a conversation* with existing scholarship.	The context and rationale for conducting research around psychological wellbeing and identity in gender affirming speech pathology context are based on theory from different disciplines. The contribution of future study findings to emerging discussions in gender affirming speech pathology care is acknowledged.	Section 1, p. 3-8; section 7, p. 31
2. Clearly articulate a research question – one that is methodologically coherent.	Can discuss refining an initially broader research question to a more specific one for the paper.	Four research questions are articulated separately and might be further refined during the research process. The qualitative research approach to address these questions is designed to be methodologically coherent.	Section 1, p. 7-8; section 2.3, p. 10-11
"Owning your perspectives"			
3. Include information on guiding theoretical assumptions and other (e.g., explanatory) theory informing the use of TA [Thematic Analysis].	Guiding (e.g., paradigmatic, ontological and epistemological) and other theory should be coherent with RTA [Reflexive Thematic Analysis].	Different theories around psychological wellbeing, identity, and speech pathology are acknowledged as influencing the study. A constructivist research paradigm applies.	Section 2.1, p. 8-9; section 2.3, p. 10
4. Report in a way that is consistent with stated theoretical assumptions throughout.	Theoretical coherence is evidenced through the use of language and concepts (e.g., around theme development, research subjectivity, data interpretation), the	Language is mainly used in consistence with the theoretical assumptions. Exceptions are made to match journal writing style. The elements of the study designed, i.e., the underlying	Throughout the study protocol (see particularly section 2.3,

[1] For the comprehensive list, see Braun and Clarke [1, suppl].

	treatment of data, and use of quality practices consistent with RTA.	paradigmatic assumptions, the data collection methods, the data analysis approach, and strategies to ensure research quality are designed to be in conceptual alignment with each other.	p. 10; section 5, p. 27-28)
5. Evidence methodological coherence/integrity in both the research and the report.	Theoretical assumptions, research questions, methods/practices of data generation, RTA, and specific orientation to RTA, purpose of research etc. all "fit together", conceptually.	Coherence between theory, study aims, research paradigm, and methods is ensured based on the research team's understanding of current literature and ongoing discussions within the team.	Throughout the study protocol (see particularly sections 1, 2.3, 2.5.3, 2.5.4)
6. Show evidence of reflexive practice.	Can discuss researcher professional or personal positioning and experience in relation to the topic, and/or participant group, and/or their role in shaping the research; use of reflexive journaling.	The research team provides a reflexivity statement that shows the cultural, professional, and disciplinary diversity of the team. The researcher's active contribution to data analysis is acknowledged. Reflexive journaling is performed by the first author during the whole research process.	Section 2.1, p. 8-9; section 2.6.1, p. 25
7. Write in a methodologically coherent style.	A first-person writing style suits RTA, as it "writes in" the researcher and contributes to situated and reflexive reporting.	First-person writing style is not applied in order to match journal writing style. Future study findings will be presented in the first-person.	---

Section 2: The Methodology

**Advice for aspects of the research report/ approach to reporting** [1, suppl]	**Guiding notes and further explanation** [1, suppl]	**Information in the study protocol**	**Addressed in section, page no.**
Participants/data items			
8. Describe selection of participants/data items.	Should include criteria for selection and/or recruitment strategies and settings.	The inclusion and exclusion criteria apply to future participating gender diverse people, speech pathologists and collaborating gender diverse advisors as outlined. Purposeful sampling is pursued predominantly. Different parties are contacted via email to recruit potential study participants.	Section 2.4, p. 11-15
9. Describe number of participants/data items; provide a rationale or explanation around	Non-positivist qualitative concepts, such as "information power" or sufficiency offer conceptually appropriate justifications for "dataset"	Nine gender diverse people and fifteen speech pathologists are planned to be included. This number is considered sufficient to support an in-depth	Section 2.4.2, p. 13-14

dataset or participant group size/composition.	or "participant group" size and composition.	understanding of the research topic for the context of the study. Three gender diverse community members are included as research collaborators/advisors.	
10. Discuss characteristics of participants/data items.	Balance the need to "situate the participant group" with participant anonymity (e.g., aggregate or report minimal demographics where appropriate).	n/a<br>(results are not reported in the study protocol)	n/a
11. Detail ethical approval and ethical code/principles followed, participant informed consent, etc.	Ethical discussion usually includes institutional ethical approval (if needed), but may include wider principles; providing research materials (participant information, consent form, etc.) in supplementary materials may be useful to support reflexive openness.	Ethical approval has been obtained. Ethical principles are applied, particularly acknowledging that gender diverse people are marginalised/ vulnerable. Members of the gender diverse community are involved as collaborators to aim for meaningful research beneficial for the gender diverse community and for speech pathologists providing gender affirming care.	Section 2, p. 8; section 2.3, p. 10-11; section 3, p. 26-27; section 4, p. 27
Dataset generation			
12. Provide some rationale for method(s) for data generation/ data item sources chosen.	Discuss why the method(s) of data generation/data source was a good fit with the research question, participant group, guiding theory, etc. If multiple data sources are used, any rationale for combination should be conceptually appropriate (e.g., crystallisation).	Sociodemographic data is collected to later contextualise what study participants share about their experiences and perspectives in the episodic interviews and focus group discussions. Episodic interviews are conducted with gender diverse people to gain a detailed insight into their personal experiences on the research topic. Focus group discussions with speech pathologists are conducted to facilitate intense discussion about the research topic.	Section 2.5.1, p. 16; section 2.5.3, p. 18; section 2.5.4, p. 20-21
13. Describe development and/or characteristics of data generation tool(s).	Include tool(s) in supplementary materials when possible; discuss piloting if used, and any changes following piloting, or during data generation.	A list of the sociodemographic data to collect as well as the episodic interview guide and focus group discussion guide are provided as supporting information. Creating the episodic interview and focus group discussion guide is supported by the gender diverse community collaborators and piloted before commencement of data collection.	Section 2.5.3, p. 19-20; section 2.5.4, p. 21-22; S3-S5 Appendix

14. Include details such as modality and/or setting of data generation, time frame, and other pertinent procedural information.	Relevant information includes: the mode of a data generation tool (e.g., video call focus groups; chat-based interviews); the context of data generation (location; timeframe) – where this doesn't compromise participant anonymity; and mode of recording interactive data generation.	The study is conducted online since study participants, collaborators and research team are not in the same location. Preliminary meetings, episodic interviews and focus group discussions take place via online videocall and are audio-recorded. Advantages and disadvantages of the online setting are outlined and briefly discussed. Interviews and discussions are audio recorded. A comprehensive list of selected online platforms and software is provided.	Section 2.2; p. 10; section 2.5, p. 16; section 2.5.1, p. 16; section 2.5.2, p. 17-18; S2 Appendix
15. Describe who conducted any interactive data generation (which author or research role), and how.	Can include what, if anything, the researcher disclosed about their personal or professional positioning or motivation; what skills and experience they brought; note researcher's relationship with participants prior to, during and after the research.	The first author's positioning, incl. motivation and skills are outlined in the reflexivity statement. The first author interacts with study participants and collaborators and shares a cultural and linguistic background with them. Study participants and first author are not known to each other before the conduction of the study. They interact multiple times during the study, i.e., during the recruitment procedure, in preliminary meetings, episodic interviews/ focus group discussions and during member reflections.	Section 2.1, p. 8-9 ; section 2.2, p. 10-11; section 2.4.1, p. 11-13; section 2.5, p. 16; section 2.6.1, p. 24-25
16. Describe the size/scope of dataset and dataset items.	Such as the range and average length for interviews/focus groups; range and average word length for textual data items.	n/a<br>(results are not reported in the study protocol)	n/a
17. Describe, and if relevant explain, any preparation of data for analysis.	Such as method of transcription of audio/video data (a transcription key can go in supplementary materials); changes and "corrections" – such as why typographical errors in written data were corrected; system for removing any identifying information; use of pseudonyms and/or data codes.	Transcripts are created following the guidelines by Braun and Clarke and Kuckartz and Rädiker. The guidelines are further specified by the research team to have a more detailed guidance for the transcription process. Study participants choose their own pseudonym.	Section 2.6.1, p. 23; section 4, p. 27
Data analysis			
18. Provide some rationale for use of RTA, and,	Any combining of RTA with other method/ologies or procedures should be	RTA is chosen to guide the process of developing themes	Section 2.6.1, p. 23-

where relevant, for combining RTA with other approaches and procedures.	warranted, rather than based on a misunderstanding of RTA, and conceptually coherent (unless clearly justified).	relevant for the research aims. The researcher has an active role in the process of meaning-making. It is expanded by different strategies to facilitate further engagement with the data, including the narrative parts, if necessary. Data source triangulation is used to synthesise data from the episodic interviews and focus group discussions. It is ensured that complementary data analysis strategies and the approach for data synthesis conceptually align with the theoretical assumptions that RTA is based on.	25; section 2.6.2, p. 25-26
19. Describe specific orientation to RTA.	Locate RTA on dimensions of inductive<>deductive and semantic<>latent.	RTA is located as mainly inductive. The analysis process is data driven and no themes are created a priori to data analysis. Both semantic and latent coding are performed.	Section 2.6.1, p. 23
20. Discuss how the researcher(s) engaged with the analytic process.	Provide a specific and situated account of the analysis process; use supplementary materials to provide a fuller account of the analytic process.	Reflexive journaling, memos, member reflections, and peer debriefing are planned for an in-depth engagement with the data.	Section 2.6.1, p. 23-25
21. Where more than one person is involved, describe who analysed the data (author or research role).	Role(s) or involvement throughout the process should be discussed; where coding was collaborative, what this involved and how differences in coding and theme development were tackled, should be included.	Data analysis is performed primarily by the first author with support from the research team, peers, community collaborators, and study participants.	Section 2.6.1, p. 25
22. Use language to describe the process and products of RTA that is coherent with the values and assumptions of RTA.	Language should convey the *active* role of the researcher(s) in “generating”, “crafting”, “constructing”, “creating”, “producing” or “developing” themes; language around themes should evokes them as *products* of a researcher-data process.	Language is chosen so that it conveys the active involvement of the researchers in the data analysis and synthesis process.	Section 2.6.1, p. 23-25; section 2.6.2, p. 25-26

Section 3: The Analysis

Note: Criteria in this section are not listed since they do not apply to the study protocol. The study protocol outlines a study plan and not study results.

Section 4: The Final Section – A General Discussion or "Conclusion"

**Advice for aspects of the research report/ approach to reporting** [1, suppl]	**Guiding notes and further explanation** [1, suppl]	**Information in the study protocol**	**Addressed in section, page no.**
Quality, evaluation and conclusions			
23. Draw analytic conclusions across themes.	Orient to the "so what" of the *overall* analysis – the "point" of the story told; this might include discussion of implications for practice and "actionable" outcomes.	n/a<br>(results are not reported in the study protocol)	n/a
24. Discuss implications or directions for future research.	Any suggestions for future research should stem from the analysis and be evidence-based (e.g., provide grounds for other groups potentially having different experiences or views) rather than generic.	Implications for the knowledge produced in the study are discussed based on the existing research and the anticipated contribution of the planned study. Directions for future studies are outlined based on the identified limitations of the planned study.	Section 7, p. 29-31
25. Use and report quality practices coherent with RTA.	Ensure evaluation of research quality deploys conceptually coherent notions, such as: member reflections; crystallisation; others serving as a critical friend/sounding board to enhance insight; reflexive journaling.	Quality practices coherent with RTA are pursued: reflexive journaling, audit trails, member reflections, and peer debriefings; contextualising future study findings is planned.	Throughout the study protocol (see particularly section 5)
26. Evaluate the research from a *Big Q* standpoint.	Such evaluation might including [sic] considering how the *specifics* of the study may have shaped the research produced (for example, the characteristics and context of the participant group/dataset; the methods and modalities for generating the data); situatedness should not be treated as a limitation.	It is discussed how the study might be shaped by its specific context, particularly the research team, future study participants, the data collection methods and the setting.	Section 7, p. 29-31
27. Include reflections on research process and practices, including researcher reflexivity.	Some consideration of the researcher(s)'s role in shaping the research and the knowledge generated is an important quality marker.	Theories around psychological wellbeing, identity, and speech pathology as well as the research team's positioning are acknowledged as influence on the study.	Section 2.1, p. 8-9

Reference

1. Braun V, Clarke V. Supporting best practice in reflexive thematic analysis reporting in Palliative Medicine: A review of published research and introduction to the Reflexive Thematic Analysis Reporting Guidelines (RTARG). Palliative Medicine. 2024;00(0):1-9. https://doi.org/10.1177/02692163241234800.
