# Supplementary material for: Gender diverse people’s psychological wellbeing and identity in the context of gender affirming speech pathology practice: A qualitative study protocol
Source: PLoS One. 2024 Nov 26;19(11):e0311402. doi: 10.1371/journal.pone.0311402 (PMC11594413; doi:10.1371/journal.pone.0311402)
Supplement: S2 Appendix — (PDF) [file pone.0311402.s002.pdf]

Gender diverse people's psychological wellbeing and identity in the context of gender affirming speech pathology practice: A qualitative study protocol

Supporting information

S2 Appendix. Online platforms and software for different phases of the study to maintain study participant data privacy.

Study phase	Purpose	Tool type	Chosen tool	Data protection arrangements
Recruitment, data collection	Schedule meetings	Online platform	Xoyondo [1]	Compliant with GDPR; study participants do not have to provide personal information [2]
Data collection, data analysis	Make videocalls, share study participant material	Online platform	ALVE Therapy [3]	Compliant with GDPR; study participants do not have to provide personal information; no video or audio recording function [4]
Data management	Create audio recording	Software	OBS Studio [5]	Study participant data not processed online
Data management	Convert OBS file format	Software	VLC media player [6]	Study participant data not processed online
Data management	Cut audio file for transcripts	Software	Audacity [7]	Study participant data not processed online
Data analysis	Create transcripts using AI	Online platform	f4x [8]	Compliant with GDPR; study participant data not used to train AI; study participant data deleted after seven days [9]
Data analysis	Edit AI-transcripts	Software	EasyTranscript [10]	Study participant data not processed online
Data management, data analysis	Manage data; analyse data	Software	NVivo [11]	Study participant data not processed online

Data protection arrangements are based on the GDPR of the European Union [12]. GDPR = General Data Protection Regulation; OBS = Open Broadcaster Software; VLC = VideoLAN Client; AI = Artificial Intelligence.

References

1. Veit Technologies. Xoyondo - Schedule meetings faster. 2023 [access date 2023 Jul 23]. Available from: https://xoyondo.com/.
2. Veit Technologies. Xoyondo - Privacy policy. 2023 [access date 2023 Jul 23]. Available from: https://xoyondo.com/privacy-policy.
3. Relearnlabs GmbH. ALVE - Interaktive Telemedizin-Plattform [ALVE - Interactive telemedicine platform]. 2023 [access date 2023 Jul 23]. Available from: https://alvetherapy.com/.
4. Relearnlabs GmbH. ALVE - Privacy policy. 2023 [access date 2023 Jul 23]. Available from: https://alvetherapy.com/wp-content/uploads/2022/07/privacy-policy-en.pdf.
5. Bailey L. OBS - Open Broadcaster Software, Version 29.1.2 [software]. 2023.
6. VideoLAN Organization. VLC media player, Version 3.0.18 [software]. 2022.
7. Audacity Team. Audacity - Free, open source, cross-platform audio software, Version 3.3.3 [software]. 2023.
8. dr. dresing & pehl GmbH. f4x 2023 Engine - f4x Automatic Speech Recognition. 2023 [access date 2023 Jul 23]. Available from: https://www.audiotranskription.de/en/f4x/.
9. dr. dresing & pehl GmbH. Privacy policy. 2023 [access date 2023 Jul 23]. Available from: https://www.audiotranskription.de/en/privacy-policy/.
10. E-Werkzeug. easytranscript, Version 2.51 [software]. 2023.
11. QSR International. NVivo Qualitative Data Analysis Software, Version 1.7.1 [software]. 2022.
12. On the protection of natural persons with regard to the processing of personal data and on the free movement of such data, and repealing Directive 95/46/EC (General Data Protection Regulation) (2016).
