# Supplementary material for: Gender diverse people’s psychological wellbeing and identity in the context of gender affirming speech pathology practice: A qualitative study protocol
Source: PLoS One. 2024 Nov 26;19(11):e0311402. doi: 10.1371/journal.pone.0311402 (PMC11594413; doi:10.1371/journal.pone.0311402)
Supplement: S3 Appendix — (PDF) [file pone.0311402.s003.pdf]

Gender diverse people's psychological wellbeing and identity in the context of gender affirming speech pathology practice: A qualitative study protocol

Supporting information

S3 Appendix. Sociodemographic data to collect from participating gender diverse people and speech pathologists.

Gender diverse people	Speech pathologists
**General information** (based on [1-4])	
Pronouns (if any)	Pronouns (if any)
Gender identity (if any)	Gender identity (if any)
Age	Age
Place of residence (rural vs. urban)	
	**Educational background** (based on [5])
	Type of speech pathology (SP) qualification
	Academic education in SP (if yes, title of the highest qualification)
	Further qualifications<br>• Area of qualification (gender affirming care, counselling, psychology or similar)<br>• Type of qualification (professional development training, university degree or other)
**Gender affirming voice modification services** (based on [4-6])	
Place of receiving gender affirming SP services<br>• Name of (former) speech pathologist	Place of providing gender affirming SP services<br>• Rural, urban, metropolitan<br>• Clinical work setting (private practice, healthcare clinic, or other)
Experience with gender affirming SP services<br>• Number of SP sessions to date (incl. date of last SP session)	Experience with gender affirming SP services<br>• Number of years of clinical work experience (incl. time frame)<br>• Average number of gender diverse clients seen over the past 12 months
Phonosurgery undertaken (if yes, type and date of surgery)	
**Miscellaneous information** (based on [7])	
How they heard about the study (community, (former) speech pathologist, or other)	Personal connection to the gender diverse community (percentage of colleagues, friends, family or gender diverse themselves)

References

1. Coleman E, Radix AE, Bouman WP, Brown GR, de Vries ALC, Deutsch MB, et al. Standards of Care for the Health of Transgender and Gender Diverse People, Version 8. Int J Transgend Health. 2022;23(sup1):S1-S259. https://doi.org/10.1080/26895269.2022.2100644.
2. Chang TK, Chung YB. Transgender Microaggressions: Complexity of the Heterogeneity of Transgender Identities. J LGBT Issues Couns. 2015;9(3):217-34. https://doi.org/10.1080/15538605.2015.1068146.
3. Jacob M, Cox S. Examining transgender health through the International Classification of Functioning, Disability, and Health's (ICF) Contextual Factors. Quality of Life Research. 2017;26:3177-85. https://doi.org/10.1007/s11136-017-1656-8.
4. Kaplan SC, Butler RM, Devlin EA, Testa RJ, Horenstein A, Swee MB, et al. Rural living environment predicts social anxiety in transgender and gender nonconforming individuals across Canada and the United States. Journal of Anxiety Disorders. 2019;66:102116. https://doi.org/https://doi.org/10.1016/j.janxdis.2019.102116.
5. Fitzgerald A. Professional identity: A concept analysis. Nursing Forum. 2020;55(3):447-72. https://doi.org/10.1111/nuf.12450.
6. Nolan IT, Morrison SD, Arowojolu O, Crowe CS, Massie JP, Adler RK, et al. The Role of Voice Therapy and Phonosurgery in Transgender Vocal Feminization. Journal of Craniofacial Surgery. 2019;30(5):1368-75. https://doi.org/10.1097/scs.0000000000005132. PubMed PMID: 31299724.
7. Hancock A, Haskin G. Speech-language pathologists' knowledge and attitudes regarding lesbian, gay, bisexual, transgender, and queer (LGBTQ) populations. Am J Speech Lang Pathol. 2015;24(2):206-21. https://doi.org/10.1044/2015_AJSLP-14-0095
