# Supplementary material for: Gender diverse people’s psychological wellbeing and identity in the context of gender affirming speech pathology practice: A qualitative study protocol
Source: PLoS One. 2024 Nov 26;19(11):e0311402. doi: 10.1371/journal.pone.0311402 (PMC11594413; doi:10.1371/journal.pone.0311402)
Supplement: S4 Appendix — (PDF) [file pone.0311402.s004.pdf]

Gender diverse people's psychological wellbeing and identity in the context of gender affirming speech pathology practice: A qualitative study protocol

Supporting information

S4 Appendix. Guide for episodic interviews with gender diverse study participants[1].

Prior to interview

- Study participants receive information about the project and the interview process via written participant information statement
- Study participants attend preliminary one-one-one meeting with interviewer
  - To have the opportunity to verbally review and confirm information about the project and the interview process (the process is explained in detail)
  - To get to know each other before the interview

PART 1 – Introduction

- Thank interviewee for their participation
- Remind interviewee of key points for interview: audio recording, style of interview questions, no obligation to answer to questions, ensure confidentiality
- Clarify interviewee's remaining questions
- Ask for interviewee's consent to start the interview

PART 2 – Interviewer guidelines

General questions and prompts (see [1, 2])

- What do you mean by…?
- (I'm not quite sure if I understand this correctly.) Could you say more about this?
- What does... mean for you?
- That sounds interesting. Can you tell me more about it?
- When was the last time that...?
- For narrations: How did it come about? – What happened then? – What did you do then? – How did it impact you?

Content-dependent questions and prompts

- Ask **additional questions** to introduce new topic dependent on content of interviewee's answer (can be left out if not suitable, i.e., if covered in interviewee's previous answer or if not applicable to interviewee's experiences)
- Ask **alternative questions** and/or give **prompts** to assist interviewee to answer a specific question (can be left out if not needed)

[1] This complete guide is the version after piloting, revision, and modification before commencing data collection.

PART 3 – Interview questions[2] (see [2, 3])

TOPIC: Identity & (psychological) wellbeing<br>(related to research question 1)	
**No.**	**Interview questions**
**1**	I would like to start with a very broad, open question and would like to invite you to think about the term 'wellbeing'.<br>• **What does "wellbeing" mean to you personally?**<br>Please take your time to think.
**2**	*(If interviewee shared narration of an episode after question 1)*<br>You talked about … (name previous aspects).<br>• **What is it that makes you feel good in such situations?**<br>• Alternative: Can you describe what makes you feel good in such situations?
*	*(If interviewee shared abstract definition after question 1)*<br>When you think back to the last weeks, months, years:<br>• **Can you think of a situation that made you feel great or, vice versa, not so good?**<br>Please pick a specific situation and tell me about it. Feel free to go into great detail.<br>• Prompt: e.g., conversation topics, what you think about yourself or how you see yourself, how you felt that day, the setting (e.g., example safe spaces, bathroom situation, laws, gender options on forms…)
**3**	Thank you for the good start to the conversation. Feeling good can be linked to identity, i.e., how you see yourself and what you think about yourself. That is a very interesting topic to me. I would like to talk to you about <u>your</u> identity, <u>your</u> self-image[3]. If you think about it:<br>• **How would you describe yourself as a person?**<br>• Alternative: What kind of person are you?<br>• Alternative: What are the most important things that define you?<br>• Prompt: e.g., age, gender identity, sexual orientation, language, profession, character traits, sense of humour, hobbies etc.

TOPIC: Self-presentation, identity & (psychological) wellbeing<br>(related to research question 1)	
**No.**	**Interview questions**
**4**	Now you've described yourself, your self-image. You mentioned... (name previous aspects). We are often not detached from other people and show ourselves to others as well.<br>• **Could you describe how you show yourself to others?**<br>• Prompt: e.g., through a certain style of clothing, a certain hairstyle, jokes, hobbies, language, voice or the way you move, etc.
**5** *	*(Additional: depending on answer to question 4; leave out, if not suitable)*<br>You've already mentioned a few things. When you think back:

[2] Questions evoking narrations are marked with an asterisk.
[3] The term 'self-image' is used in a lay manner as a synonym for 'identity' to give the interviewee an idea of how identity is understood.

	• **Do you remember a concrete situation in which it was particularly important or, vice versa, not important at all to present yourself through... (name previous aspects)?**<br>Please tell me about it.
**6** *	*(Additional: depending on answer to question 4; leave out, if not suitable)*<br>We talked about how you show yourself to others. You mentioned… (name previous aspects). When you think back to the last few weeks, months, years:<br>• **When was the last time you were perceived by others the way you would like to be perceived?**<br>Please tell me about a specific situation.
**7** *	*(Additional: depending on answer to question 4; leave out, if not suitable)*<br>• **And vice versa? Has it ever not worked out that others perceived you the way you want them to?**<br>Please tell me about a specific situation.

TOPIC: Vocal patterns, identity & (psychological) wellbeing
(related to research question 1)

**No.**	**Interview questions**
**8** *	*(Additional: depending on answer to question 5; leave out, if not suitable)*<br>Thank you so far. We'll take a closer look at the "<u>how</u>" of presenting yourself. Think back again:<br>• **Please tell me about a situation where you felt uncomfortable with your voice (or name previous aspects)?**<br>What kind of situation was that? Feel free to tell me about it.
**9** *	*(Additional: depending on answer to question 5; leave out, if not suitable)*<br>• **And vice versa? Please tell me about a situation wherein you felt comfortable with your voice (or name previous aspects)?**<br>What kind of situation was that? Feel free to tell me about it.
**10**	• **Do you have an image of how your "ideal" voice (or name previous aspects around voice) should be like or does this not exist?**
**11**	*(Additional: depending on answer to question 10; leave out, if not suitable)*<br>• **If yes, please describe this voice (or use interviewee's term/wording) to me.**
**12**	• **Would you say that you already have your ideal voice (or use interviewee's term/wording) or not?**
**13**	*(Additional: depending on answer to question 10; leave out, if not suitable)*<br>• **Assuming you had your "ideal" voice, your "dream voice" (or use interviewee's term/wording), what would change for you in your everyday life? Or would it not change anything?**
**14**	We talked about your identity, your self-image and you had described yourself as... (name previous aspects), now we also talked about your ideal voice.<br>• **Would you say that it works well for you to express your identity through your voice or does that not work that well?**
**15**	*(Additional: depending on answer to question 14; leave out, if not suitable)*<br>• **When do you notice that?**

TOPIC: Experiences with speech pathology practices (related to research question 2)	
**No.**	**Interview questions**
**16** *	We have now talked about voice (or use interviewee's term/wording), about showing yourself to others. You are currently seeing a speech pathologist (or: You used to see a speech pathologist).<br>• **Please tell me about your experiences with speech pathology.**<br>Start with the first time you went to a speech pathologist and how it went from there.
**17**	If you think back to the very beginning:<br>• **What did you hope to achieve with the help of a speech pathologist?**<br>• Alternative: What did you hope that a speech pathologist could support you with?
**18**	If you think back and think of today, what do you think:<br>• **Did you get the support through speech pathology that you wanted or was there something missing?**<br>• Alternative: To what extent would you say that you have achieved what you wanted?
**19**	*(Additional: depending on answer to question 18; leave out, if not suitable)*<br>• **What support would you have wished for?**
**20**	*(Additional: depending on answer to question 18; leave out, if not suitable)*<br>• **What remained unresolved or what else would you have liked to have achieved?**

TOPIC: Effects of speech pathology practices (related to research question 2)	
**No.**	**Interview questions**
**21** *	Your day-to-day life is also very interesting to me, which is one of the reasons why we are here. When you think back to the time since you first went to see a speech pathologist:<br>• **Have you had any experiences in your everyday life where you noticed any change since you have seen a speech pathologist or rather not?**<br>Tell me about a situation. You are welcome to go into great detail.<br>• Prompt: e.g., when interacting with others, when using your voice, on the phone, when talking to strangers (e.g. in the supermarket), when talking to friends or family etc.
**22** *	We talked about your ideas about your voice or about ways of expressing yourself. When you think back, what do you think:<br>• **Has speech pathology changed anything about how you see or evaluate your voice, the way you speak, intonate etc., or rather not?**<br>Could you talk about a specific experience?<br>• Prompt: e.g., how you perceive your voice, how you evaluate it, what you expect from your voice etc.
**23**	*(Additional: depending on answer to question 21; leave out, if not suitable)*<br>Let's go back to your self-image. Please think about the last few weeks, months, years.<br>• **Do you think that speech pathology has changed something about how you see yourself or not?**

**24**	*(Additional: depending on answer to question 23; leave out, if not suitable)*<br>• **If yes, how does that show, when do you notice it?**
**25**	*(Additional: depending on answer to question 21, 23; leave out, if not suitable)*<br>We are now coming to the last part of the interview, and I would like to go back to the beginning: wellbeing. At the beginning you said what wellbeing means to you.<br>• **Does or did seeing a speech pathologist have any influence on how good you feel overall or is that rather not the case?**
**26**	Thank you. Now my last question for today:<br>• **What does the ideal speech pathology intervention look like for you personally?**

PART 5 - Debriefing	
**No.**	**Interview questions & follow-up questions**
**27**	Thank you for sharing your experiences and perspectives with me. That was all from my part. Let me briefly summarise what we talked about: You talked about... (give summary of the interview).<br>• **Is there anything else we did not talk about that you would like to add or comment on?**

- Point out how to find help in case interviewee experiences distress: local helplines, contact interviewer
- Thank interviewee for their participation

References

1. Mack N, Woodsong C, Macqueen K, Guest G, Namey E. Qualitative Research Methods: A Data Collector's Field Guide. North Carolina: Family Health International; 2005.
2. Flick U. Doing Interview Research: The Essential How To Guide. Los Angeles: SAGE Publications; 2021.
3. Flick U. Episodic Interviewing. In: Bauer MW, Gaskell G, editors. Qualitative Researching with Text, Image and Sound. London: SAGE Publications; 2000. p. 75-92.
