# Supplementary material for: Gender diverse people’s psychological wellbeing and identity in the context of gender affirming speech pathology practice: A qualitative study protocol
Source: PLoS One. 2024 Nov 26;19(11):e0311402. doi: 10.1371/journal.pone.0311402 (PMC11594413; doi:10.1371/journal.pone.0311402)
Supplement: S5 Appendix — (PDF) [file pone.0311402.s005.pdf]

Gender diverse people's psychological wellbeing and identity in the context of gender affirming speech pathology practice: A qualitative study protocol

Supporting information

S5 Appendix. Guide for focus group discussions with participating speech pathologists[1].

Prior to focus group discussion

- Study participants receive information about the project and the focus group discussion process via written participant information statement
- Study participants attend preliminary one-one-one meeting with facilitator
  - To have the opportunity to verbally review and confirm information about the project and the focus group discussion process (the general process is explained; more detail is provided before the discussion)
  - To get to know each other before the focus group discussion

PART 1 – Introduction

- Thank focus group participants for their participation
- Remind participants of key points for discussion: audio recording, style of discussion facilitation, no obligation to contribute to the discussion, ensure confidentiality
- Explain the procedure for the discussion in more detail: role of the facilitator (asks questions, not actively involved in discussion), role of the participants (discuss with each other, encouraged to ask each other questions)
- If group consists of participants with different speech pathology educational backgrounds: clarify which term to use to refer to their profession when facilitating the discussion
- Clarify participants' remaining questions
- Ask for participants' consent to start the discussion

PART 2 – Facilitator guidelines

General questions and prompts (see [1, 2])

- What do you mean by…?
- (I'm not quite sure if I understand this correctly.) Could you say more about this?
- Do you have an example for this?
- What do the others think? Do you agree or do you have different opinions or experiences?
- Think of you experiences: Do the others have something to add here?

Content-dependent questions and prompts

- Use **additional questions** to introduce new topic dependent on content of the discussion (can be left out if not suitable, i.e., if covered in previous discussion or if not applicable to participants' experiences)
- Give **prompts** to assist participants to talk about a certain topic (can be left out if not needed)

[1] This complete guide is the version after piloting, revision, and modification before commencing data collection.

PART 3 – Focus group discussion prompts (see [3])

TOPIC: Introductions<br>(to create a welcoming space)	
**No.**	**Discussion prompts**
**1**	As you know, what you all have in common is that you work or have worked with trans, gender diverse, non-binary clients. Please introduce yourselves to each other and then pass on to the next person. I will pick the first person to start. My question now is:<br>• **Where do you currently work and what else would you do on a (name weekday and time) if you do not participate in a focus group?**

TOPIC: General clinical work with gender diverse clients<br>(related to research question 3, 4)	
**No.**	**Discussion prompts**
**2**	Thank you for the nice introductions. I would like to continue with an open, general question for you. You can also answer openly, it can go into any direction. Think back to your work with trans and gender diverse people so far:<br>• **What do you work on with trans or gender diverse people?**<br>Feel free to share your experiences with each other.
**3**	You now have already mentioned a lot, for example… (name previous aspects).<br>• **What is particularly important to you in your work in gender affirming care?**<br>Please share your thoughts with each other.
**4**	• **As speech pathologists, how do you see your purpose in working with trans and gender diverse people?**

TOPIC: Speech pathology practices & identity<br>(related to research question 3, 4)	
**No.**	**Discussion prompts**
**5**	Let's look at the topics of wellbeing and identity in trans and gender diverse people.<br>• **Would you agree that - among other things - the self-image[2] or how someone experiences their own identity has an impact on your clients' wellbeing or would you disagree?**
**6**	*(Phrasing depending on the previous discussion.)*<br>(Some of) You agreed that self-image or how your client experiences their own identity plays a role for their wellbeing. We know that wellbeing is a goal of healthcare for gender diverse clients.<br>• **Does this mean that speech pathologists should work with gender diverse clients on their self-image or identity, or who would be responsible for this?**<br>Please share your thoughts.
**7**	• **Would you say that you work on identity or self-image or are you rather not? And if so, how do you do that?**<br>Feel free to talk about that.
**8**	*(Additional: depending on discussion about question 7; leave out, if not suitable)*

[2] The term 'self-image' is used in a lay manner as a synonym for 'identity' to give the focus group participants an idea of how identity is understood.

	Now you've mentioned more functional elements, i.e., breathing, posture, voice, intonation, articulation, etc. (name previous aspects).<br>• **In addition to these more functional elements, are there other approaches that you use to work on self-image or identity or not?**<br>Could you think of some examples?
9	• **What determines whether you work with your clients on their self-image or identity?**<br>• Prompt: What prevents you from working in this area?<br>• Prompt: What would help you to work in this area?

**TOPIC: Speech pathology practices & wellbeing** (related to research question 3, 4)	
**No.**	**Discussion prompts**
**10**	Let's take a look at wellbeing.<br>• **Would you say that you work on your clients' wellbeing or not? If yes, how do you do that?**
**11**	Thank you so far. Let's move on to the next question.<br>• **Were there ever moments in your work when you wondered whether what you were doing was helpful for your clients or was that rather not the case?**
**12**	*(If participants shared experiences that indicate a general "yes" to question 11)*<br>• **What other methods or tools would you have liked to have?**
	(If participants shared experiences that indicate a general "no" to question 11)<br>• **What has helped you in particular in your work with trans and gender diverse people?**
**13**	We are now at the end of the discussion. You have discussed a lot of different things.<br>• **When you think back to what you discussed today, what has been the most important thing for you personally?**<br>Someone can start. Please pass on to the next person after you have answered.

**PART 5 - Debriefing**	
**No.**	**Discussion prompts**
**14**	Thank you for sharing your experiences and perspectives with me. That was all from my part. Let me briefly summarise what you have discussed: You talked about... (give summary of the focus group discussion).<br>• **Is there anything else that you would like to add or comment on?**

- Thank participants for their participation

References

1. Mack N, Woodsong C, Macqueen K, Guest G, Namey E. Qualitative Research Methods: A Data Collector's Field Guide. North Carolina: Family Health International; 2005.
2. Krueger RA. Designing and Conducting Focus Group Interviews. 2002.
3. Krueger RA, Casey MA. Focus groups: A Practical Guide for Applied Research. Thousand Oakes: SAGE Publications; 2015.
