# Supplementary material for: Gender diverse people’s psychological wellbeing and identity in the context of gender affirming speech pathology practice: A qualitative study protocol
Source: PLoS One. 2024 Nov 26;19(11):e0311402. doi: 10.1371/journal.pone.0311402 (PMC11594413; doi:10.1371/journal.pone.0311402)
Supplement: S7 Appendix — (PDF) [file pone.0311402.s007.pdf]

Gender diverse people's psychological wellbeing and identity in the context of gender affirming speech pathology practice: A qualitative study protocol

Supporting information

S7 Appendix. Plain language summary.

Some gender diverse and transgender people do not feel good about the sound of their voice. Additionally, some gender diverse and transgender people might not feel comfortable in themselves overall. Feeling comfortable is connected to a person's self-image, or to what a person thinks about themselves. Healthcare aims to support gender diverse and transgender people to feel more comfortable. Speech pathologists can help gender diverse and transgender people to work on the sound of their voice so that it feels right for them. This can make gender diverse and transgender people feel more satisfied and confident with their voice. However, some gender diverse and transgender people might still not feel comfortable overall. Gender diverse and transgender people can work on their self-image so they can think about themselves in a more positive way. This work could be a useful part of speech pathology support as well. It could help gender diverse and transgender people feel more comfortable overall. Speech pathologists must feel responsible to help their clients to feel more comfortable in themselves.

This study aims to find out more about what gender diverse and transgender people, who want to change the sound of their voice, think about themselves and about feeling comfortable. Gender diverse and transgender people will be interviewed individually about these topics. Additionally, this study wants to find out about what speech pathologists think about and do when helping gender diverse and transgender people to feel comfortable and good about themselves. Speech pathologists will discuss these topics in groups with other speech pathologists. Interviews and discussions will be interpreted to find out about common and different topics that come up. A small group of gender diverse and transgender people supports this study with their advice. Study findings will be communicated to speech pathologists and gender diverse and transgender people.

Study results help speech pathologists to better support their gender diverse clients.
