# Supplementary material for: Gender diverse people’s psychological wellbeing and identity in the context of gender affirming speech pathology practice: A qualitative study protocol
Source: PLoS One. 2024 Nov 26;19(11):e0311402. doi: 10.1371/journal.pone.0311402 (PMC11594413; doi:10.1371/journal.pone.0311402)
Supplement: Attachment — Submitted filename: Response to Reviewers.docx [file pone.0311402.s008.docx]

**Rebuttal letter**

**PLOS ONE reviewers’ comments**

**for manuscript**

| **Manuscript title** | Gender diverse people’s psychological wellbeing and identity in the context of gender affirming speech pathology practice: A qualitative study protocol |
| --- | --- |
| **Manuscript ID** | PONE-D-23-43876 |

16 June 2024

Dear Dr. Carels,

Thank you for inviting us to submit a revised version of our manuscript. We are grateful to you and the reviewers for the time and effort put in to providing us with such helpful feedback and guidance for our revision of the manuscript we submitted.

Please find below four tables containing our responses to each of the reviewers’ comments as well as a list of additional modifications we have made to enhance our manuscript. Please note that the significant changes requested in the revised manuscript have led to an increase in the number of pages of our manuscript and to additional supporting information that we have now provided.

We would be grateful if you would consider our revised manuscript for publication in PLOS ONE. We are looking forward to receiving a response in due course.

Kind regards,

Julia Tanase (corresponding author)

# 1. **Reviewers’ comments to the authors – Reviewer #1**

| **Reviewer’s comments** | **Authors’ reply** |
| --- | --- |
| Thank you for this well-planned study protocol which is supposed to be used to increase knowledge about trans peoples´ well-being in the context of speech pathology practice for gender-affirming voice and communication training. The manuscript is well-written. The purpose of the planned study is clear and will fill an evident knowledge gap. Only a few studies have been performed in the area previously. Abstract – fine! Background – the references referred to are relevant and up-dated and well described. | Thank you for your thought-provoking feedback. We appreciate your thorough review of our manuscript and your valuable comments. You will find our reply to each of your comments below. The pages, sections, and lines we refer to in our replies are those in the revised unmarked manuscript version. |
| Page 3, line 51- Please define the term cisgender | We have provided a clearer explanation of this term when we describe the difference in depression, anxiety, etc. between gender diverse people and “cisgender people who do identify with their gender presumed at birth”, see line 53. |
| Page 4, line 72. Please expand on the meaning of ‘identity fluid’ so that the concept is not misinterpreted as ‘gender fluid’. | We have elaborated more on identity to avoid confusion and stated that “This ongoing, individual and interindividual meaning-making means that a person’s identity is in constant change and therefore mutable”, see lines 88-89. |
| Page 5. Lines 120-122. This question may also be related to the context which the SP work within. In many European countries, the SP is part of an inter-professional gender team (including psychiatrists and psychologists) which together works on a ‘holistic’ approach. This is probably quite different from other clinical contexts such as private practicing SPs. | We agree with the point you raise (referring to the original statement that “at present, it is unknown whether speech pathologists who provide gender affirming care view their role in this way and whether they already apply more holistic approaches to their clinical work.”).  To support our study aims, we have added a new section to elaborate more on the clinical professionals and explain that how a clinician sees their role in supporting gender diverse people might depend on the context in which they practice their profession. We have given an example to explain that the clinical setting in which speech pathologists provide gender affirming care (i.e., interdisciplinary healthcare clinics vs. single discipline settings with peer speech pathologists) might influence whether they feel responsible for addressing client psychological wellbeing. We have also stated that it is unclear how the professional setting influences speech pathologists’ mindset regarding client support. The new section can be found in lines 148-158. |
| Page 6. Lines 127-128. Based on the SP context the first question could be addressed in a broader sense to the gender team, and not only to the SP. | We agree that it will be important to explore this question related to other professions in future research. However, due to the lack of research around identity and psychological wellbeing in gender affirming speech pathology clinical care, other professions are not our current focus.  To address your point, we have stated that our research could be a good starting point for similar research around identity and psychological wellbeing in gender affirming healthcare, see lines 189-192. We have also suggested in our discussion that focussing on speech pathology in gender affirming healthcare is a limitation of the study and that studies with other professions are warranted to gain insights on the research topic from a different perspective and in a different context, see lines 701-705. |
| Page 6. Lines 135-137. “The research questions are based on the research team’s current knowledge”. Then it is important to understand a bit more about the research team’s clinical context (private, hospital, part of a gender team) and education? | Thank you for this valid point. We agree that the information originally given in the reflexivity statement in one of the later sections, is not sufficient and that the reader needs more guidance and information here. To address this, we have:   - removed the sentence you are referring to in order to avoid confusion. - given the reader more information about the background of the research team. We have added   - The clinical setting in which members of the research team provided gender affirming care, see lines 206-207 and 215-216.   - The multidisciplinary educational background (speech pathology, sociology, psychology) and research interests (identity in speech pathology) of the different members of the research team, see lines 213-215. |
| Methods: At this stage the study is not yet completed, and you still work on the procedure for the data collection and analysis. Therefore, it would not be possible to replicate the study based on the study protocol. | Thank you for the comment. As we are unsure as to what exactly you are referring to, we would like to address your comment in two different ways:  In case your feedback refers to ensuring that the study provides enough detail to be replicated  Even though the study has not been completed yet, we would like to provide as much detail as possible about the planned study via this study protocol. We do this to support others who would like to take a similar methodological approach and/or would like to use the theoretical conceptualisations of psychological wellbeing and identity for their own research in this area. We have added this as an aim of the study protocol, see lines 186-192.  To provide more detail about our study, we have added the interview guide and focus group discussion guide as supporting information, see S4 Appendix and S5 Appendix. This has led to editing changes in the manuscript:   - We have referred to the full interview and focus group discussion guides as supporting information, see lines 460-461 and lines 502-503. - To match the layout of the interview guide in the supporting information with the example questions in the manuscript, we have edited Table 2 in the manuscript, see p. 20, Table 2. We have not made any changes to the content of the table. - We have listed the interview and focus group discussion guide as supporting information, see lines 767-769.   Further, we have explained the value of the study protocol in the discussion section. We have now stated that the study protocol is important to meet quality criteria of qualitative research (credibility, transferability) and that it provides great detail on the planned study, see lines 692-696.  In case your feedback refers to the fact that the study is not yet finished as qualitative research is a dynamic process  We have outlined the value of the study protocol and have stated that comparing the study protocol with later publications on the study will demonstrate the dynamic process of qualitative research, see lines 181-185. |
| How many participants (clients and SPs) do you plan to include? | We plan to include nine gender diverse people, and fifteen speech pathologists for three focus group discussions. We have now included this information in section 2.4.2 “Sampling”. We have also stated that we anticipate this number to be sufficient to support an in-depth understanding of the research topic, but that the sample will be extended in case data analysis shows the need to gather more information. We explain this for both the gender diverse people and the speech pathologists in lines 324-327 and 332-334 respectively.  We plan to include three community advisory group members and now have added this information as well, see lines 335-337. |
| Page 7, line 151. Specify the ‘gender affirming SP contexts’. | Thank you for this. To highlight that we focus on speech pathologists who work clinically in the area of gender diversity we have:   - specified that “‘clinical’ refers to any healthcare setting providing direct client support, i.e., private practices, or healthcare clinics.”, see lines 246-247. - added the word “clinical” when the context of gender affirming care might be unclear, see lines 170, 246. We also have added the word “clinical” for new writing added in the manuscript, whenever the context might be unclear. |
| Page 7. Lines 154-155. Please motivate why you will use different methods for collecting data: individual interviews for clients and focus groups for SPs. | We agree that this is important information. We have given a short explanation in the section you are referring to (2.3 “Methodology and study design”). We have now stated that episodic interviews will be conducted to gain a detailed insight into gender diverse people’s personal experiences on the research topic and that focus group discussions with speech pathologists will be conducted to facilitate intense discussion about the research topic, see lines 252-255.  In the data collection sections (2.3.5 and 2.3.4), we have now also elaborated more clearly on why we chose each method.  For the episodic interviews, we now have explained that we chose this method   - to create a safe space to talk about sensitive topics (psychological wellbeing, identity). - to target both episodic and abstract knowledge. We have highlighted here that the advantage of targeting episodic knowledge in interviews is that it provides insight into people’s meaning-making in their day-to-day life. - to have an interview form that is particularly open to both negative and positive narrations of situations.   We have explained this in lines 437-452.  For the focus group discussions, additional to the aim of evoking both diversity and agreement about the research topics, we now have contrasted that the group process can support participants to explore their perspectives on a certain topic as opposed to one-on-one settings. We have specified that speech pathologists’ actions in supporting gender diverse clients’ psychological wellbeing and identity are rarely discussed in speech pathology literature. We have explained this in lines 485-489. |
| It seems good and meaningful to collect data in two parts to be able to stop and reflect on the data collecting process before continuing the full study. | Thank you for your kind feedback. |
| Page 7, line 177. A . I missing after practices. | Thank you for noticing this. We have corrected the interpunction, see line 208. |
| Page 8, line 188. You have a hypothesis “that speech pathologists working in this area might find it challenging to think of themselves as clinicians supporting clients’ PWB”. Please describe what makes supporting transgender clients in their PWB so different from supporting other clients in the SP care in their PWB (such as clients with cancer, neurodegenerative diseases etc). Do not most SPs work with councelling to give psychological support to patients with communication disorders? SPs must be well-prepared but need special education about transgender clients and minority stress of course if they are not experienced with the client group. | Thank you for this valuable thought. We would like to reply to the different points you raise individually:  Supporting speech pathology clients through counselling  We agree that counselling should be part of any speech pathology approach with clients. However, we found that this is not necessarily the case. We now have briefly outlined that some speech pathologists might be hesitant to use counselling in their work. Clinicians might be unsure about their scope of practice and might even feel conflicted when counselling clients, as shown in research in the field of aphasia rehabilitation, see lines 159-164.  Education about gender diverse clients and minority stress  To address your thought, we now have stated that additional to professional training in gender affirming voice modification approaches, a full understanding of a gender diverse client’s individual challenges and resources in life is crucial when providing gender affirming care that aims to support client psychological wellbeing, see lines 121-123. |
| Page 8. Line 196. Please reflect on the advantages and disadvantages with on-line interviews. | Thank you for this comment. We have included the information you requested as follows:   - In section 2.4.3 “Recruitment procedure and inclusion”: We have noted the advantage of including study participants and collaborators from different geographical areas in Germany when conducting an online study, see lines 362-363. - In section 2.5 “Data collection”: We have outlined that online data collection might impact study participants’ engagement during data collection, but is overall not inferior to in-person data collection, see lines 374-376. - In section 7 “Discussion”: We have added people’s access to the internet as another limitation of the study, see line 717. |
| Page 9. You have decided to exclude SPs who are “widely known” to avoid hierarchical power dynamics in the focus groups. If those SPs are also highly experienced in the area of gender affirming SP practices, are you not worried to miss important from those? Why do you not offer individual interviews also with the SPs as with the clients? And what is the definition of “widely known” and who will decide this? There seems to be a risk of bias if this is the investigator’s decision. | Thank you for your questions. We would like to address them individually as follows:  Data collection method with the speech pathologists  We chose focus group discussions over interviews to evoke both diversity and agreement about the research topic and to support speech pathologists to discuss the research topics. We now have rephrased our rationale to make it clearer that the focus group process can support participants to explore their perspectives on a certain topic as opposed to one-on-one settings, see lines 485-489.  Definition of the exclusion criterion “widely known” and risk of bias   - We have changed the wording to speech pathologists who are likely to be considered “experts” by peers. We have now also specified that people are considered as experts when they are “widely known through their publications in gender affirming clinical practices”, see lines 295-298. - To match the wording in the manuscript, we have changed the wording in the table for the inclusion and exclusion criteria to “Likely to be considered experts by peer speech pathologists”, see p. 12-13, Table 1, column “speech pathologists”, column “exclusion criteria”.   Missing information if speech pathologists “widely known” in the area are excluded:   - Thank you for this thought. To address this, we have added that we accept this disadvantage in favour of avoiding potential conflicts of interest in the focus group discussions, see lines 299-301. - Further, we now have suggested that future studies could conduct one-on-one interviews with experts in the field without risking hierarchical power dynamics within peer focus groups, see lines 729-733. |
| Page 11, line 250. So, recruitment is already completed? Later it says that data collection is completed, confusing. | Yes, by now, recruitment and data collection have been completed. However, when we submitted early January 2024, recruitment and data collection were still in progress.  We agree that this might be confusing for the reader and therefore consulted with the editor. The editor’s advice was to indicate how many study participants and collaborators are expected to be included rather than providing detail about who has already been recruited. Since this information has been added to section 2.4.2 “Sampling” (see one of you earlier comments and our reply above), this would not add any new information. Therefore, we decided to remove this section from the study protocol paper. |
| Page 11, line 251 speech pathologists - SP. Earlier in the text you have written the abbreviation SP and later speech pathologist. Please be consistent. | We use “SP” as an abbreviation for “speech pathology” as a profession. We have added this information, see lines 96-97. We use no abbreviation for “speech pathologists”.  In order to be in line with common editing conventions, we spell out “SP” as “speech pathologists” in the tables we provide. |
| Page 12, line 264. You write that the SPs “are known to the investigator”. Isn’t this a risk of bias? | The part you are referring to was worded in a confusing way. To address this, we have made the following changes:   - We now have clarified that “speech pathologists known to the research team will not be included in the study but will be asked to pass on relevant study information to potential study participants”, see lines 353-354. - To be clear that the exclusion criterion of “being known” applies to all study participants and to the whole research team, not only the first author, we rephrased it in Table 1. The exclusion criterion now reads as “Knows members of the research team before study participation”, see p. 12-13, Table 1, column “exclusion criteria” for gender diverse people and speech pathologists. |
| Page 12, Line 267. This is also written for the AG group “known to the investigator personally”. Risk of bias? | Thank you for this comment. We do not consider it generally problematic to collaborate with gender diverse people previously known to the research team in the AG group. However, we have specified:   - that gender diverse people previously known in a non-professional context will be excluded from collaboration, see lines 302-303 and p. 13, Table 1, column “Advisory Group”, column “exclusion criteria”. - the different roles of the advisory group and study participants. We have stated that “people who know the team from a professional context can be included in the AG since they are collaborators in this project as opposed to study participants”, see lines 303-305. |
| Page 12-13. Line 281-284. It is somewhat unclear if the investigator will meet the SPs individually before the focus group. | We have now specified that the first author will meet with each gender diverse person and each speech pathologist individually, see lines 376-379. |
| Page 13, line 294-295. “Speech pathologists will receive an explanation of the focus group process just before the start of the focus group discussion”. Unclear if this is taken place in the individual meeting with the SP. | We have specified that speech pathologists will receive a general explanation of the focus group discussion process in the preliminary meeting and a more detailed explanation before the discussion starts. We have also explained that we chose this approach since speech pathologists are considered less vulnerable than gender diverse people, see lines 388-391. |
| Page 13, lines 296-207 “providing gender affirming SP services”. What more information will you collect from the SPs? I would assume that information about working environment (working alone, working with colleagues, with a interprofessional gender team), years working with transgender clients, with patients with voice disorders, and what kind of diagnoses. What kind of education do the SPs have, BA? MSc? Advanced courses in SP? Have they been participating in national/international conferences within the transgender area? This is important to describe since SP educations in Europe differ a lot (some are even not at a university level). This is important if ou think that your results can be generalized. | Thank you for this comment. We have made the following changes to address this:   - We have added a rationale for collecting sociodemographic data and have stated that this helps to “contextualise each study participant’s experiences and perspectives in relation to the study aims.”, see lines 392-394. - We have given different examples for the sociodemographic data we collect: gender diverse people’s information on their gender identity and the number of gender affirming SP sessions attended and speech pathologists’ educational background in speech pathology and their clinical work setting, see lines 394-396. - We have provided a comprehensive list of the sociodemographic data we plan to collect in the supporting information, see S3 Appendix, have referred to this list in the manuscript, see line 396-397, and have listed it as supporting information, see line 765-766. |
| Page 13, line 303. A . is missing after the parenthesis. | Thank you for noticing this. We corrected the interpunction, see line 403. |
| Page 14. 315-317 “Notes will be taken during data collection, e.g., about participants’ choice of wording or their non-verbal communication”. Please clarify if this will be done from the audio-recordings after the sessions, or from video-recordings that seems necessary if non-verbal communication will be described. It is written earlier that audio-recordings are carried out. Please clarify. | We have specified that the first author will take notes during the interviews and discussions, see lines 416-417.  We have moved the information about the type of non-verbal communication that will be noted further up, so that the section reads as “The first author will take notes during the interviews and discussions, e.g., about participants’ choice of wording and their non-verbal communication, e.g., if participants nod or use other gestures instead of words.”, see lines 416-418.  Since we consider this type of non-verbal communication as rather basic (as opposed to emotional facial expressions, for example), we do not consider video recordings to be necessary. Furthermore, from clinical experience in gender affirming care, some people might not feel comfortable with video recordings. Video recordings might have also influenced study participants’ behaviour in the interview/discussion. |
| Page 14, line 328. “..reflections about the investigator’s behaviour”. Who will do those reflections? | We have specified that the reflections are the first author’s own reflections. We have further specified the kind of reflections, i.e. the first author’s “reflections about her behaviour as interviewer or facilitator, impressions of the interaction between the first author and the study participants including potential influences of the online format of data collection, and initial thoughts about conversation contents.”, see lines 430-433. We have also specified that these notes will be used for further reflections on the data collection process and for later data analysis, see lines 433-434. |
| Table 2. Items 15 and 18 are leading questions (which you wrote earlier in the text that should be avoided). | Thank you for raising this concern. We do not consider these to be leading questions. We have now specified that the interviewee will be asked open questions and have given an example. Therefore, we have stated: “Instead, the interviewee will be asked open questions so that they do not feel like they have to respond in any particular way (e.g., “Did you get the support through speech pathology that you wanted or was there something missing?”).”, see lines 467-470. |
| Status of the project Page 23, line 517: Data collection is planned to be completed by 29 February 2024. Please update this sentence. Did you already complete data collection? On page 11 you write that “recruitment period is anticipated take place from 1 June 2023 to 29 February 2024”. Recruitment and data collection is not the same. | Yes, by now, recruitment and data collection have been completed. As outlined in one of our earlier replies, we agree that this might be confusing for the reader, and we therefore consulted with the editor and have decided to remove this section from the study protocol paper (see reply above). We have also removed the sentence you are referring to in order to avoid confusion. |
| Discussion You have lifted relevant issues regarding methodological issues and limitations. Maybe add a sentence about if you think that you results can be generalized. | Thank you for your comment. We use different strategies to make research findings valuable for other contexts, i.e., to contribute to transferability. To specify this, we have made changes in section 5 “Research quality”: We have now explained that we use contextualisation of study findings and purposeful sampling for this purpose, see lines 654-656. We have added that contextualisation of study findings means to provide great detail on the study context to transparently show how the study findings are generated, see lines 648-650. We also have explained that purposeful sampling is performed “to achieve variability in the sample of study participants”, see line 657.  Further, to provide a more comprehensive discussion of our study, we have now added a section that states that the study participants who are planned to be included are a potential limitation of the study. Here, we have also stated now that despite this limitation, purposefully aiming for a variety in the sample of gender diverse people will contribute to a diversity of perspectives and experiences and therefore to transferability of study findings. The newly added section in the discussion part can be found in lines 706-716. |
| References: Check consistency regarding capital letters (or not) in the titles of the articles. Ref 10 and 17 – are there more information? | Thank you for picking up on those details. We will address your points individually.  Inconsistency of capital letters  We agree that this is inconsistent. We have double-checked with the general rules for the citation style PLOS ONE uses (PLOS ONE uses Vancouver Style). It is recommended to cite the title of a reference as it appears in the original document (for example, see <https://www.ncbi.nlm.nih.gov/books/NBK7282/#A32422> for citation of journal articles or <https://www.ncbi.nlm.nih.gov/books/NBK7271/#A34227> for citation of books). This is why our citations of titles are inconsistent. We also double-checked the reference style of already published study protocols through PLOS ONE in the past 5 years. They show this stye of title citation as well.  More information about references   - We added more information to reference 10 and 17, see p. 34, section 10 “References”, reference number 10 and 16. - Additionally, we have updated the doi format for all references with doi number, so it shows up as links for easier retrieval of references, see p. 33-43, section 10 “References” |
| The results from this study, when finished, will be useful for many SPs working in similar clinical contexts as the one in the study. I widh you the best for completing the study. | Thank you again for your thorough review and your detailed feedback to help us improve our manuscript. |

# 2. **Reviewers’ comments to the authors – Reviewer #2**

| **Reviewer’s comments** | **Authors’ reply** |
| --- | --- |
| Is it ok for a registered report to be published AFTER data collection? Line 250 says recruitment takes place June 2023 to February 2024. | Thank you for pointing this out. By now, recruitment and data collection have been completed. However, according to the journal guidelines, a study protocol can be published after data collection, as long as it is submitted before the completion of data collection (see <https://journals.plos.org/plosone/s/submission-guidelines#loc-study-protocols>). |
| The proposed qualitative study is justified and well-designed. It is scientifically rigorous and ethically conscientious. | Thank you for your thought-provoking feedback. We appreciate your thorough review of our manuscript and your valuable comments. You will find our reply to each of your comments below and above. The pages, sections, and lines we refer to in our replies are those in the revised unmarked manuscript version. |
| The methodology is feasible and detailed, although without all the interview questions it cannot be replicated as well. Could the full list of questions be provided as supplemental material? | To address this and make our research process more transparent, we made the following changes:   - We have provided a comprehensive list of the sociodemographic data we plan to collect in the supporting information, see S3 Appendix, have referred to this list in the manuscript, see line 396-397, and have listed it as supporting information, see line 765-766. - We have added the interview guide and focus group discussion guide as supporting information, see S4 Appendix and S5 Appendix. This has led to editing changes in the manuscript:   - We have referred to the full interview and focus group discussion guides as supporting information, see lines 460-461 and lines 502-503.   - To match the layout of the interview guide in the supporting information with the example questions in the manuscript, we have edited Table 2 in the manuscript, see p. 20, Table 2. We have not made any changes to the content of the table.   - We have listed the interview and focus group discussion guide as supporting information, see lines 767-769. |
| Without the German transcripts being made available, it may be wise to have more than 2 people conducting the analysis part of this study. As diverse as the research team is, the meaning-making at this stage is critical and limited by who is able to participate in it. | Thank you for your comment. We now have outlined that even though the first author will be analysing the data mainly by herself, this process will be supported using different strategies. Strategies include member reflections with study participants and peer debriefings with the research team, peer qualitative researchers, and the gender diverse collaborators in this study. Transcripts in German will be translated into English for additional discussions with the whole research team. Diverse perspectives are acknowledged using these strategies. We have now outlined this in the manuscript, see lines 563-575.  Further, we have now given a rationale for why transcripts will be analysed in German first and translated into English later: to avoid potential language restrictions in English, see lines 568-569. Earlier in the manuscript we have stated that the first author will analyse the data, see line 563 and that the first author is a native German speaker, see lines 208-209.  We now have also outlined that the first author will perform reflexive journaling to document critical reflections during data analysis as a crucial part of reflexive thematic analysis, see lines 576-581. |
| Four research questions are provided but the hypotheses are not clear enough to be tested. Due to the nature of this exploratory qualitative study, the hypotheses do not need to be testable. However, it would be helpful to explicitly state the implied hypotheses or assumptions found in the research questions in order to justify the line of questioning and build the results into a testable hypothesis or theory. This seems feasible based on the provided background information. My understanding of the rationale would suggest that the authors hypothesize that…  1. gender-diverse people seeking SLP support (can/can’t) experience their identity as positive. Those experiencing it as positive are more likely to include sources of support in their stories. 2. gender-diverse people seeking SLP support (do/don’t) identify changing vocal patterns AND managing their distress regarding communication as valuable. They identify misgendering and other factors in the minority stress model as not valuable. 3. speech pathologists (do/ don’t) see their role as supporting voice as well as PWB and perspective on identity. 4. speech pathologists report providing support ranging from … (referrals to mental health practitioners to providing counseling). In other words, it is hypothesized that the outcome data will align with the empirical and best practice literature described in the introduction and provide further detail. | Thank you for this thought-provoking comment.  You outlined in your comment that we hypothesise that the outcome of our data will align with the current literature. We would like to emphasise that given the lack of current research on the research topic (psychological wellbeing and identity in gender affirming speech pathology care) and given the research approach we have chosen, including a data-driven analysis, we are open to any type of outcome in our study. However, we have acknowledged different theories as influential on our research. We now have emphasised this and have added that “linking these theories as outlined in the background section of this protocol have influenced the design of this study.”, see lines 224-225. |

# 3. **Reviewers’ comments to the authors – Reviewer #3**

| **Reviewer’s comments** | **Authors’ reply** |
| --- | --- |
| Thank you for the opportunity to review this study protocol.  Study protocols for qualitative research projects are as important to publish as those of quantitative research efforts. However, the dynamic approach to information gathering used in the qualitative research method makes a pre-hoc description of the study design a challenge. This particular study focuses on the psychological wellbeing and identity of clients of gender affirming voice training and have opted to use a reflective approach to analysis of the material. Community participation is built in in the planned research. I have a couple of reflections that I would like the authors to consider before starting the research. | Thank you for your thought-provoking feedback. We appreciate your thorough review of our manuscript and your valuable comments. You will find our reply to each of your comments below. The pages, sections, and lines we refer to in our replies are those in the revised unmarked manuscript version.  To address your comment about the challenge of describing a dynamic approach of qualitative research in a study protocol, we now have stated that qualitative research is a dynamic process. We have now further elaborated that “comparing the study protocol with later publications on the study will demonstrate this dynamic process.”, see lines 181-185. |
| First, the study focuses on the clients' identity and wellbeing, and while the study seems well equipped to provide a well balances presentation of the clients' identity, I am not sure that wellbeing is captured adequately. At least not all applicable aspects of wellbeing I can think of. With an answer to the question of "how" the study protocol will allow elucidation of the study aims, I cannot se a strong reason why it should be published as a study protocol. As it may not be of use to others (the readers) wanting to throw light on the same or related aspects in a different context. The authors need to strengthen the link between wellbeing, according to all applicable definitions, and the planned discussions with participants, in particular with the transgender participants. | Thank you for your valuable reflections. We would like to address each point of your comment individually in the following:  Usefulness of the study protocol for others  We have added a section to specify that the study protocol could be useful to others as methodological orientation and as a theoretical basis to further investigate topics such as psychological wellbeing and identity to deepen the understanding of these topics for gender affirming healthcare services, see lines 186-192.  Definition of psychological wellbeing  We have now elaborated more on the hedonic and eudaimonic approach to conceptualise psychological wellbeing and have given a rationale as to why we chose the eudaimonic conceptualisation of psychological wellbeing for this study. We have elaborated that the eudaimonic conceptualisation of psychological wellbeing seems appropriate to understand gender diverse people’s psychological wellbeing since it takes into account that people may have to negotiate challenges throughout their lifespan in order to give meaning to such events. This meaning-making process is closely connected to how a person sees themselves and to their psychological wellbeing. At the end of this added information, we have referred to the following paragraphs that will elaborate more on gender diverse people’s challenges and their perspective on themselves to link this new section to the already existing content in the theoretical background. This whole section on psychological wellbeing can be found in lines 54-67.  Stronger link between theories about psychological wellbeing and data collection methods  We have made the following changes to be able to provide a clearer link between theory and chosen data collection methods:   - Additional to the elaborated definition of psychological wellbeing (see lines 54-67), we have added more information around non-disclosure of identity and authentic presentation of gender identity in the theoretical background. We have elaborated that gender diverse people might not disclose their gender diverse identity to others to avoid external stressors, which can further contribute to negative psychological wellbeing, see lines 71-75. We have further added that feeling authentic and openly presenting one’s experienced gender identity to others can act as a resource for psychological wellbeing, see lines 78-79. - We have now outlined that the data collection methods used are based on the research aims and current literature   - For the episodic interviews, we have added that literature informing the study covers the topics of psychological wellbeing, identity, gender minority stress, (gender) self-presentation, and gender affirming speech pathology practices. We have also listed the different topics covered in the interview guide: (1) identity and psychological wellbeing, (2) self-presentation, identity and psychological wellbeing, (3) vocal patterns, identity and psychological wellbeing, (4) experiences with speech pathology practices, and (5) effects of speech pathology practices. We have added this in lines 453-461.   - For the focus group discussions, we have added that literature informing the study covers the topics of psychological wellbeing, identity, gender minority stress, and speech pathology practices in gender affirming care. We have also listed the different topics covered in the focus group discussion guide: (1) General clinical work with gender diverse clients, (2) speech pathology practices and identity, and (3) speech pathology practices and psychological wellbeing. We have added this in lines 496-503. - For more transparency, we have provided our interview guide and focus group discussion guide as supplemental material, see S4 Appendix and S5 Appendix, have referred to them in the manuscript, see lines 460-461 and lines 502-503, and have listed them as supporting information, see lines 767-769. |
| Please also note that there are recent works that you have not included in the preparation of the study protocols that brings forward the fears of transgender clients when entering into training  Holmberg, J., Linander, I., Södersten, M. & Karlsson, F. (2023). Exploring Motives and Perceived Barriers for Voice Modification: The Views of Transgender and Gender-Diverse Voice Clients. Journal of Speech, Language, and Hearing Research, 66(7), 2246–2259. [https://doi.org/10.1044/2023_jslhr-23-00042](https://url.au.m.mimecastprotect.com/s/irYMC6X1kRtggWKlFBBb-p?domain=doi.org) | Even though we cited Holmberg et al. (2023) already in the original version of our manuscript in different contexts, the article was not cited in the context you suggest here.  As you have suggested, to support our study aims, we have now included the findings of Holmberg et al. (2023) about the experienced distress during speech pathology sessions and its impact. We have now explained that “an intense engagement with their own vocal patterns might result in gender diverse clients experiencing distress, which can limit their capacity to engage in SP [speech pathology] even though they wish to do so.”, see lines 112-115.  Additionally, based on the clinical implications of Holmberg et al. (2023), we have added creating a supportive, accepting speech pathology setting and reflecting on gender norms as possible ways to support client psychological wellbeing, see lines 125-127. |
| And a more expansive discussion of subgroups in the transgender speakers, with regards to gender identity, that the authors need to re-consider (while one is cited)  Azul, D. & Hancock, A. B. (2020). Who or what has the capacity to influence voice production? Development of a transdisciplinary theoretical approach to clinical practice addressing voice and the communication of speaker socio-cultural positioning. International Journal of Speech-Language Pathology, 22(5), 559–570. [https://doi.org/10.1080/17549507.2019.1709544](https://url.au.m.mimecastprotect.com/s/Sn7sC71ZlLu00Rkju2eNqA?domain=doi.org)  Merritt, B. (2023). Speech beyond the binary: Some acoustic-phonetic and auditory-perceptual characteristics of non-binary speakers. JASA Express Letters, 3(3), 035206. [https://doi.org/10.1121/10.0017642](https://url.au.m.mimecastprotect.com/s/_kPqC81Zm6uAAkR8hEbB_U?domain=doi.org) | We would like to address these suggestions individually:  Including findings from Azul and Hancock (2020)  In addition to the existing citation of Azul and Hancock (2020), we have now added information on supporting client psychological wellbeing based on the clinical implications that Azul and Hancock (2020) make, i.e., developing responses to instances of misgendering as one additional approach in gender affirming speech pathology practices, see line 126.  However, we note that the study by Azul and Hancock (2020) does not refer to specific gender identity groups.  Including findings from Merritt (2023)  The suggested study by Merritt (2023) has been a good starting point for acknowledging the subgroups in the gender diverse community. We have now outlined that some gender diverse clients do not want to be perceived as cisgender female or male, whereas others do. We have further elaborated that this shows that client needs are highly individual and reflects the heterogeneity of the gender diverse community, see lines 99-103. For this statement, we have cited a study that was used in the theoretical background of the study by Merritt (2023). We have not included study results from Merritt (2023) since their study focusses on speaker’s acoustic characteristics and the link to listeners’ perception and gender-attribution, which was not a focus of our study.  In our original version of the manuscript, we have acknowledged the different subgroups in the gender diverse community in our theoretical background when we write about emerging gender affirming speech pathology practices that are inclusive of different gender identities, see lines 129-131. We also acknowledge subgroups in our sampling by aiming for a diverse sample of gender diverse study participants, see lines 312-318. |
| In this work. How barriers to voice training comes into may interact with both the client's and SLPs views is not clearly addressed in the study protocol. And with wellbeing not being well defined, there is no strong presence of participant validation of transcripts or conclusions drawn, and there is not standardized tool used to further elucidate the level of stress perceived, the study protocol is too likely to not provide a strong basis for a description of high validity | We would like to address the different points you raise individually:  Barriers to voice training  We deliberately chose not to focus only on barriers for gender affirming speech pathology care or on negative experiences of gender diverse people for the following reasons:   - Psychological wellbeing and particularly identity in gender affirming speech pathology practices have not been explored extensively to date, especially in a broader sense beyond gender presentation or vocal patterns, see section 1 “Background”, p. 3-7. To gain a comprehensive understanding of these research topics, we focus on both positive and negative experiences of gender diverse people. - It is important to us to minimise emotional distress, especially for the gender diverse study participants. Therefore, we chose a data collection method that is open to both positive and negative experiences. We have added this information about the data collection method, see lines 450-452. Further, we ask for both positive and negative experiences as elaborated in lines 462-470. - To counter a deficit-oriented discourse, we aim to explore the research topic in a more comprehensive way. We have elaborated on this and have now added an additional brief note to communicate that this is also reflected in our study aims, see lines 262-265 for the added note.   Link between theoretical background and conclusion for data analysis  To strengthen our theoretical background, we have now elaborated more on the hedonic and eudaimonic approach to conceptualise psychological wellbeing: We chose the eudaimonic conceptualisation of psychological wellbeing for this study as this conceptualisation seems appropriate to understand gender diverse people’s psychological wellbeing since it takes into account that people may have to negotiate challenges throughout their lifespan in order to give meaning to such events. This meaning-making process is closely connected to how a person sees themselves and to their psychological wellbeing, see lines 54-67.  The theoretical assumptions around psychological wellbeing and identity could be helpful to link the data analysis to existing research. As the data analysis process will be data driven, we are also open to the possibility that different theoretical models will be needed in interpreting the data.  Using a standardised tool to assess study participants’ level of stress   - Because our goal was to gain an in-depth understanding of gender diverse people’s and speech pathologists’ experiences in a gender affirming speech pathology clinical setting, we focussed on qualitative methods. This approach seems suitable based on current research on the research topic, see p. 3-7, section 1 “Background”. - However, we have now discussed using standardised tools in section 7 “Discussion”. We have stated that based on findings from qualitative research (including this study), future studies could apply both qualitative and quantitative methods to explore the research topic. For instance, results from research using standardised tools to investigate gender diverse people’s psychological wellbeing and experienced identity could be later used to develop a holistic assessment in gender affirming speech pathology care. We have now discussed this in lines 738-743.   Participant validation  Following a constructivist research paradigm, we do not use strategies like member reflections for validation, instead we want aim for a deeper understanding of the data. We will use member reflections to acknowledge diverse perspectives in the data analysis process. We have now elaborated more on this, see lines 563-567. Furthermore, strengthening the theoretical background on psychological wellbeing and identity could support the data analysis process as explained above.  Validity of the study  We expect a high validity as we anticipate gaining a deeper understanding of our study participants’ experiences and perspectives on the research topic. We ensure this through a flexible research approach, i.e., the semi-flexible format of the interviews and focus group discussions (see lines 462-472 and 504-507 respectively) as well as the data-driven approach to data analysis (see lines 536-537).  Furthermore, to meet the criterion of credibility, i.e., the plausibility of qualitative research findings, we will apply different strategies: reflexivity throughout the whole study, member reflections, peer debriefing and contextualising the study findings, see section 5 “Research quality”, lines 646-650. |
| Please provide a stronger link between the research questions asked, the planned interview components of both the transgender and SPL participants, a plan for how the SPL and transgender findings will be contrasted or linked, and stronger processes aiming for a grounding the analyses with the participants and the broader community of clients. | Thank you for your comment. If this comment refers to the general quality of this study, we have now provided a filled-in checklist for the Reflexive Thematic Analysis Reporting Guidelines in the supporting information, see S1 Appendix. We have now referred to this checklist in the manuscript, see lines 198-199 and have listed it as supporting information, see lines 761-762.  We would also like to address the different points you raise individually:  Link between the research questions and the chosen methods of data collection  As elaborated in one of our previous replies to one of your comments, we made the following changes to provide a clearer link between our theory, including the derived research questions, and our data collection methods:   - In the theoretical background: we have elaborated on the definition of psychological wellbeing (see lines 54-67), non-disclosure of identity (see lines 71-75), and authentic presentation of gender identity (see lines 78-79). - In the data collection part: We have now outlined that the data collection methods used are based on the research aims and current literature. We have elaborated on this for the episodic interviews (see lines 453-461) and the focus group discussions (see lines 496-503). - In the supporting information: We have provided our interview guide and focus group discussion guide as supplemental material, see S4 Appendix and S5 Appendix.   How study results from gender diverse people and speech pathologists are contrasted  To address this, we have added a new section outlining that we plan to compare study results from the group of gender diverse study participants and participating speech pathologists by using data source triangulation, see lines 521-525 and 584-593. In the discussion part, we discuss that the synthesis of the study findings will provide valuable insights on the similarities and differences regarding the needs of gender diverse people and speech pathologists and that these insights may be useful for developing strategies to improve SP services for gender diverse people, see lines 734-738.  Grounding the analysis with the participants and broader community  Thank you for this comment. We are not quite sure what grounding refers to here.   - If grounding the analysis with the participants refers to participant validation: In line with a constructivist view, we will use member reflections to acknowledge diverse perspectives in the data analysis process and to gain a deeper understanding of the data. We have now elaborated more on this, see lines 563-567. - If grounding the analysis with the broader community refers to generalisation of the study results: Different steps are planned to make research findings valuable for other contexts, i.e., to contribute to transferability. To specify this, we have made changes in section 5 “Research quality”: We have now explained that we use contextualisation of study findings and purposeful sampling for this purpose, see lines 654-657. We have added that contextualisation of study findings means to provide great detail on the study context to transparently show how the study findings are generated, see lines 648-650. We also now have explained that purposeful sampling is performed “to achieve variability in the sample of study participants”, see lines 657.   Further, to provide a more comprehensive discussion of our study, we have now added a section that states that the study participants who are planned to be included are a potential limitation of the study. Here, we have also stated now that despite this limitation, purposefully aiming for a variety in the sample of gender diverse people will contribute to a diversity of perspectives and experiences and therefore to transferability of study findings. The newly added section in the discussion part can be found in lines 706-716.  If this comment refers to something else, please let us know, so that we can address it. |

4. **Changes made in addition to reviewers’ comments**

We would like to communicate the following changes we have made in addition to the reviewers’ comments:

| **Type of change** | **Location of change** |
| --- | --- |
| Minor editing changes:   - If incorrect: correction of orthography, interpunction, prepositions, and conjunctions - Changed numbering of different sections - Changed wording for clarification, e.g., the use of “first author” or “research team” instead of “investigator” - Added sub-headings for coherence and consistency with the added content - Note that references in brackets have been updated during the revision process | Throughout the manuscript |
| Revised abstract to match added contents in theoretical background of the manuscript | Abstract, lines 17-31. |
| We decided for the Reflexive Thematic Analysis Reporting Guidelines (RTARG) instead of the COREQ checklist. The RTARG were published after submission of the manuscript and are guidelines specific to the data analysis approach we use. These guidelines serve as checklist to transparently communicate the strategies used to ensure research quality. We have referred to these guidelines in the manuscript. | 2 Materials and methods, lines 198-199  6 Dissemination, line 663-664 |
| In the methodology section, we have added the information that data will be analysed using Reflexive Thematic Analysis to be consistent with the more detailed information on data collection that we now proved in the revised manuscript. | 2.3 Methodology and study design, lines 258-259. |
| We have rephrased the section about the size of the focus groups to make it clearer that we chose small focus groups to make it more likely for every participant to engage in the online discussion. | 2.5.4 Focus group discussions with speech pathologists, lines 489-491 |
| Since the manuscript revision required further refinement of our research quality criteria, we revised this section. | 5 Research quality, lines 636-659 |
| We moved the section with the list of supporting information before the reference list. This is to match the PLOS template for study protocols instead of the PLOS manuscript body formatting guidelines. We made the decision to change this based on the layout of study protocols that have already been published in PLOS ONE. | 9 Supporting information, lines 761-772. |
| Due to the revised content in the manuscript, the reference list was updated.  Note that the numbering of the references in the marked version and the unmarked version of the manuscript differ. This is because the referencing software (EndNote) still lists some references that were excluded in the tracked changes version of the manuscript. Since the tracked changes have been accepted for unmarked version, EndNote updates the reference list.  The reference list in the unmarked version of the revised manuscript is the correct reference list. | 10 References, p. 33-43 |
| The new supporting information files we have added led to an extensive list of supporting information. To improve clarity and coherence, we re-named the supporting information files with continuous numbering as follows: “S1 Appendix”, “S2 Appendix”, “S3 Appendix” and so on (similar to this published article: [https://journals.plos.org/plosone/article?id=10.1371/ journal.pone.0282925](https://journals.plos.org/plosone/article?id=10.1371/journal.pone.0282925)) | See each file of the supporting information: S1-7 Appendix |
| We referred to the supporting information throughout the manuscript using the updated numbering | 2 Materials and methods, line 199  2.2 Setting of the study, line 241  2.5. Data collection, lines 396-397, 460-461, 502-503  3 Collaboration with the gender diverse community, line 609  6 Dissemination, line 671-672 |
| We revised the supporting information files to match the revised numbering of the files as outlined above. | S2 Appendix, S6 Appendix, S7 Appendix |
| We revised the flowchart for the contributions of the Advisory Group and research team to match the revised wording in the manuscript. | S6 Appendix |
| We revised plain language summary to match added contents in theoretical background of the manuscript | S7 Appendix |
