# Supplementary material for: Gender diverse people’s psychological wellbeing and identity in the context of gender affirming speech pathology practice: A qualitative study protocol
Source: PLoS One. 2024 Nov 26;19(11):e0311402. doi: 10.1371/journal.pone.0311402 (PMC11594413; doi:10.1371/journal.pone.0311402)
Supplement: Attachment — Submitted filename: Response to Reviewers.docx [file pone.0311402.s009.docx]

**Rebuttal letter**

**PLOS ONE reviewers’ comments**

**for manuscript**

| **Manuscript title** | Gender diverse people’s psychological wellbeing and identity in the context of gender affirming speech pathology practice: A qualitative study protocol |
| --- | --- |
| **Manuscript ID** | PONE-D-23-43876 |

Thank you for inviting us to submit a revised version of our manuscript. We are grateful to the editors and the reviewers for the time and effort put in to providing us with such helpful feedback and guidance for the second revision of the manuscript we submitted.

Please find below four tables containing our responses to each of the reviewers’ comments as well as a list of additional minor changes we have made to enhance (formatting, wording, added missing information, updated literature) our manuscript.

We would be grateful if you would consider our revised manuscript for publication in PLOS ONE. We are looking forward to receiving a response in due course.

Kind regards,

Julia Tanase (corresponding author)

# 1. **Reviewers’ comments to the authors – Reviewer #1**

| **Reviewer’s comments** | **Authors’ reply** |
| --- | --- |
| Dear authors,  thank you for your excellent work to revise the manuscript with additional information so that the readers can fully comprehend your well-planned study protocol. The additions and changes have further improved the quality of the manuscript.  I just have a few comments/questions: | Thank you for your very kind and encouraging comment and your great help with improving the first version of our manuscript. Many thanks for your further comments and questions on the current version of our manuscript. We, once again, appreciate your thorough review. You will find our reply to each of your comments below. The pages, sections and lines we refer to in our replies are those in the revised unmarked manuscript version. |
| Lines 354-359. All participants should have had at least 5 SP sessions for inclusion. Is this true for participants PFAB and PMAB? I do not read German and I cannot interpret the content in reference 71. Many people, also experts, do not expect that participants AFAB also need SP-training. It would be good with some clarification here. | Thank you for this comment. To address this, we have made the following changes:  SP with people PFAB:  Thank you for pointing out that some important information needs to be included. We now have added the information that gender diverse people may feel a discomfort with their vocal patterns and seek professional support form a speech pathologist and have emphasised that “This can be the case for any gender diverse person, e.g., transfeminine, transmasculine, or non-binary people, even for people whose vocal patterns may have changed through gender affirming hormones.”, see lines 98-102.  Minimum number of SP sessions:  We have clarified that the literature does not specify a particular minimum number of SP sessions for gender diverse clients except for people aiming to present as female, which is a reported minimum number of five. We have stated that we assume that this number is transferable to any gender diverse person, regardless of whether their vocal patterns have changed through gender affirming hormones. We then have concluded that therefore, the inclusion criterion of having attended at least five SP sessions will apply to any gender diverse person interested in participating in the study, see lines 297-303. |
| Line 810 you name other professions beyond SP. Wouldn't it be appropriate to also mention psychotherapy? | Thank you for this comment. We have now made the following changes:   - Since there are few studies in psychology that explore PWB and identity in gender diverse people, we have included some more information in the background section and have stated that “Research in psychology is beginning to show that creating positive narratives around gender diverse identities can have positive effects on gender diverse people’s PWB.”, see lines 140-141. - To avoid evoking the impression that there are no studies in psychology on PWB and identity in gender diverse people, we have changed the statement in the part you are referring to slightly. We now have stated that future studies could centre disciplines beyond SP, such as, psychology, endocrinology, or occupational therapy and that the already existing literature in psychology could be a valuable resource when planning such studies, see lines 721-725. |
| Lines 816-817 "Furthermore gender diverse people who have had negative experiences with SP in the past might not want to participate in a study conducted by a speech pathologist." What do you base this assumtion on? Do you think this is common? Is the sentence really needed or can it be phrased differently? | We have phrased this differently and have added references to support our statement. We have explained that due to experiences of discrimination or harassment in healthcare settings, some gender diverse people might not want to participate in a study conducted by a healthcare professional, such as a speech pathologist, see lines 730-732. |
| I found that S6 in the appendices could be a presented as a figure as it was in the original version of the manuscript but I leave this to you to decide what you find the best. | Thank you for this comment. We have double-checked the file format for figures (.tif). Unfortunately, this format does not allow a very high resolution of the figure. Since the PDF format has a higher resolution and is allowed as a format for supporting information files, we decided to leave the file in a PDF format. |
| Good luck finalizing the manuscript. I really look forward to read about the results! | Thank you again for your feedback and your help to improve our manuscript. We really appreciate your time and effort. |

# 2. **Reviewers’ comments to the authors – Reviewer #3**

| **Reviewer’s comments** | **Authors’ reply** |
| --- | --- |
| I am very happy with the revisions. The description now clearly highlights the added benefit of the publication of the study protocol, and presents the rationale and design decisions in a clear and convincing manner. | Thank you for your very kind and encouraging comment and for your great help with improving the first version of our manuscript. We really appreciate your time and effort. |

# 3. **Reviewers’ comments to the authors – Reviewer #4**

| **Reviewer’s comments** | **Authors’ reply** |
| --- | --- |
| A really thoughtfully written protocol on a fascinating sounding study.  All the revisions made thus far have strengthened the paper. | Thank you for your very kind and encouraging comment. Many thanks for your further comments on the current version of our manuscript. We appreciate your thorough review. You will find our reply to each of your comments below. The pages, sections, lines, and reference numbers we refer to in our replies are those in the revised unmarked manuscript version. |
| However, I would like to suggest the inclusion of Braun and Clarke's 2006 and 2020 papers on the practical aspects of undertaking either a reflexive thematic analysis or a codebook thematic analysis, as both are equally useful and valid but are the not the same process.  Braun, V. and Clarke, V. 2020. One size fits all? What counts as quality practice in (reflexive) thematic analysis? Qualitative Research in Psychology, 18, 328–352. [https://doi.org/10.1080/14780887.2020.1769238](https://url.au.m.mimecastprotect.com/s/qgxdCGvmG5U7Rp21HktytqDLTe?domain=doi.org).  Braun, V. and Clarke, V. 2006. Using thematic analysis in psychology. Qualitative Research in Psychology, 3, 77–101.  At the moment, the analysis plan reads like a mix of both so a little more clarity is needed. | Thank you for pointing out this important literature and for noticing that clarity is needed here. To provide more clarity, we have made the following changes in the section about data analysis:  Differentiation between codebook TA and RTA:  To highlight that RTA is the analysis method we use and to avoid the impression that we use codebook TA, we made the following changes:   - Minor changes to the wording (see lines 549-563): We now have stated that - the aim of RTA is to develop themes, - the first author creates meaning, - RTA helps guiding the process of developing themes, - codes are based on collected data, - codes are clustered together to generate themes - Additional information: We have explained that themes will not be created a priori and analysis will be data driven with the first author actively constructing themes. We have further explained that this process will be shaped by the first author’s reflections about the context in which data analysis takes place (as a core element of RTA) and have referred to our reflexivity statement earlier in the manuscript, see lines 550-554. - After further discussion within the author team, we have reviewed our implementation of member reflections. With the changes we now have made, we would like to emphasise that member reflections will not be used to validate data but will be used in a way that is in accordance with RTA. Therefore, we have made the following changes:   - We have described that when implementing member reflections, we will send study participants “the first author’s ideas about potential initial themes constructed from the interviews or focus group discussions” and will ask study participants about their reflections on these ideas. Due to this change, we have also changed the phase in which we will be using member reflections (phase 3 instead of 1 or 2). These changes can be found on p. 25, in Table 4, row “Member reflections”.   - In the section about research quality, we have slightly changed the wording. Where we wrote about credibility, we now state that strategies like member reflections will be used to gain more insight into the data, see lines 664-665.   Inclusion of further references:  We have now cited the 2020 paper by Braun and Clarke (2021 in the reference version we have) you suggested. However, even though we see the value of the 2006 paper, we decided to cite more recent work by Braun and Clarke in this section instead, particularly since Braun and Clarke partially criticise their own 2006 paper. We have cited the following literature by Braun and Clarke instead: their 2021 paper as suggested (reference number 104, cited multiple times more now), their 2022 book (reference number 52, as cited previously), and their 2023 paper (reference number 103, as cited previously). This literature contains the information needed to make the changes in the manuscript as you suggested. The reference changes we have made can be found on p. 23-24, in section 2.6.1. Note that the changes made to the references are unfortunately not visible in the track changes version of the manuscript since this is not possible with the referencing software (EndNote) we use. |
| Also this sentence in the manuscript is confusing: "Since RTA is not seen as suitable for the analysis of narratives [49], the data analysis approach will be expanded to enable analysing narratives in the interviews or focus group discussions". What type of 'narrative' do you mean? Reflexive thematic analysis is often used (and used well) with different interview narratives in an array of qualitative studies. This needs to be clarified. | Thank you for this thought-provoking comment that prompted further discussion within the author team. We have made the following changes in the manuscript:  Definition of narratives:   - In the data collection section, where we write about narratives in episodic interviews, we have clarified what we mean by “narratives”. We have stated that narratives comprise both a story (i.e., the sequence of events) and a plot (i.e., the speaker’s attitudes, perceptions and motives) related to an episode, see lines 458-460. - For clarity reasons, we added another brief description of narratives in the data analysis section later in the manuscript, see p. 25, Table 4, row “Memos”.   RTA and the analysis of narratives:  Instead of stating that RTA is not suitable to analyse narratives, we have clarified how and why we will use the strategies we have listed as complementary strategies for RTA. We have made the following changes:   - To highlight that an in-depth engagement with the narrative parts of the data is optional, we have clarified that, if a more in-depth engagement with participants' stories is needed, memos will be created based on questions to engage with the narrative parts (story and plot of an episode) of the data, see p. 25. Table 4, row “Memos”. - Since in our previous version of the manuscript, it read as if we would use memos only for the narrative parts of the data, we have now clarified that memos, member reflections and peer debriefing aim to facilitate further engagement with the data and to support the first author to construct meaningful themes. We have referred to Table 4 for more detail, see lines 571-575. |
| Overall though, this is an excellent study and I look forward to the protocol being published and learning more about the findings in the future. | Thank you again for your feedback and your help to improve our manuscript. We really appreciate your time and effort. |

4. **Changes made in addition to reviewers’ comments**

We would like to communicate the following changes we have made in addition to the reviewers’ comments:

| **Type of change** | **Location of change** |
| --- | --- |
| Minor editing changes:   - If incorrect: correction of orthography, interpunction, prepositions, conjunctions, and tenses - Minor changes to wording or phrasing for clarity or due to the changes required for the manuscript revision - Changed spelling of terms to be consistent - Note that reference numbers in brackets have been updated during the revision process | Throughout the manuscript |
| We have changed the wording to comply with American English. Therefore, we have changed the word “might” into “may” throughout the manuscript.  We have not made any changes to the spelling of words that are spelled differently in American vs. Australian English, so we use Australian English spelling in the manuscript. If you would like us to change the spelling as well, please let us know. | Throughout the manuscript |
| Wording in the research questions: We changed wording in research questions 3 and 4 from “perspective on” to “experience of” identity to be consistent with the phrasing in research question 1. | 1 Background, lines 184 and 186. |
| Added literature to back-up statements in the manuscript and removed incorrect citations.  Due to the revised content in the manuscript, the reference list has been updated. | Throughout the manuscript  10 References |
| Added information in the Reflexive Thematic Analysis Reporting Guidelines (RTARG) in the supporting information. Some changes are related to the changed contents of the revised manuscript, some additions have been made as information was missing in the original filled-in RTARG. | S1 Appendix |
| Formatted bullet points in S4 and S5 for consistent spacing of bullet points. | S4 Appendix  S5 Appendix |
